# Supplementary material for: Myeloid-Specific STAT3 Deletion Aggravates Liver Fibrosis in Mice Fed a Methionine- and Choline-Deficient Diet via Upregulation of Hepatocyte-Derived Lipocalin-2
Source: Cells. 2025 Sep 29;14(19):1522. doi: 10.3390/cells14191522 (PMC12524133; doi:10.3390/cells14191522)
Supplement: Supplementary file 1 [file cells-14-01522-s001.zip › cells-3856673-supplementary.pdf]

Table S1. List of primers used for genotyping and RT-PCR

Gene name	Primer sequences (mouse)
Floxed *STAT3*	Forward 5′ CCTGAAGACCAAGTTCATCTGT 3'
	Reverse 5′ CACACAAGCCATCAAACTCTGGTCTCC 3'
LysM-cre	Forward 5′ GTGAAACAGCATTGCTGTCACT 3'
	Reverse 5′ GCGGTCTGGCAGTAAAAACTA 3'
Lcn2	Forward 5′ TCCTCAGGTACAGAGCTACAA 3'
	Reverse 5′ GCTCCTTGGTTCTTCCATACA 3'
Gapdh	Forward 5′ AAATGGTGAAGGTCGGTGTG 3'
	Reverse 5′ CATGTAGTTGAGGTCAATGAAGG 3'

Table S2. List of primary antibodies

Antibody	Company	Catalog No.	Dilution	Application	Source
STAT3	Cell signaling	#9139	1:200	WB	Mouse
pSTAT3	Cell signaling	#9145	1:1,000	WB	Rabbit
SREBP-1c	Santa Cruz	sc-13551	1:1000	WB	Mouse
ACC	Cell signaling	#3676	1:1000	WB	Rabbit
FAS	Cell signaling	#3189	1:1000	WB	Rabbit
SCD1	Cell signaling	#2438	1:1000	WB	Rabbit
CD36	Novus	NB400	1:1000	WB	Rabbit
GS	Santa Cruz	sc-74430	1:5000	WB	Mouse
ACOT2	Proteintech	15633-1-AP	1:3000	WB	Rabbit
PEX11A	Novus	NBP2-94259	1:1000, 1:100	WB, IHC	Rabbit
Catalase	Abcam	Ab209211	1:5000	WB	Rabbit
LC3B	Cell signaling	#83506	1:1000	WB	Mouse
p62	Sigma	P0067	1:1000	WB	Rabbit
LAMP1	Abcam	ab62562	1:1000	WB	Rabbit
TFEB	Proteintech	13372-1-AP	1:1000	WB	Rabbit
LCN2	R&D	AF1857	1:1000, 1:200	WB, IHC	Goat
Arg1	Abcam	ab60176	1:1,000	WB	Goat
MMP9	Abcam	ab38898	1:1,000	WB	Rabbit
Lumican	Abcam	ab168348	1:1000	WB	Rabbit
α-SMA	Abcam	ab32575	1:1,000	WB	Rabbit
NF-kBp65	Cell signaling	#6959	1:1000	WB	Mouse
IL-6	MYBiosource	MBS3007753	1:1000	WB	Rabbit
HMGB1	Abcam	ab18256	1:3,000	WB	Rabbit
Galectin-3	Santa Cruz	sc-23938	1:1,000	WB	Rat
HO-1	Enzo	ADI-SPA-895	1:1,000	WB	Rabbit
NQO-1	Abcam	ab34173	1:1,000	WB	Rabbit
GPX-4	Santa Cruz	sc-166570	1:1,000	WB	Mouse
4-HNE	Abcam	ab46545	1:1,000	WB	Rabbit
PCNA	Cell signaling	#13110	1:1000	WB	Rabbit
p21	Abcam	ab188224	1:1000	WB	Rabbit
Ki67	Cell signaling	#12202	1:100	IHC	Rabbit
GAPDH	Cell signaling	#97166	1:1000	WB	Mouse
p84	Abcam	Ab487	1:1,000	WB	Mouse
β-actin	Sigma	A5441	1:1,000	WB	Mouse

WB, western blot; IHC, immunohistochemistry

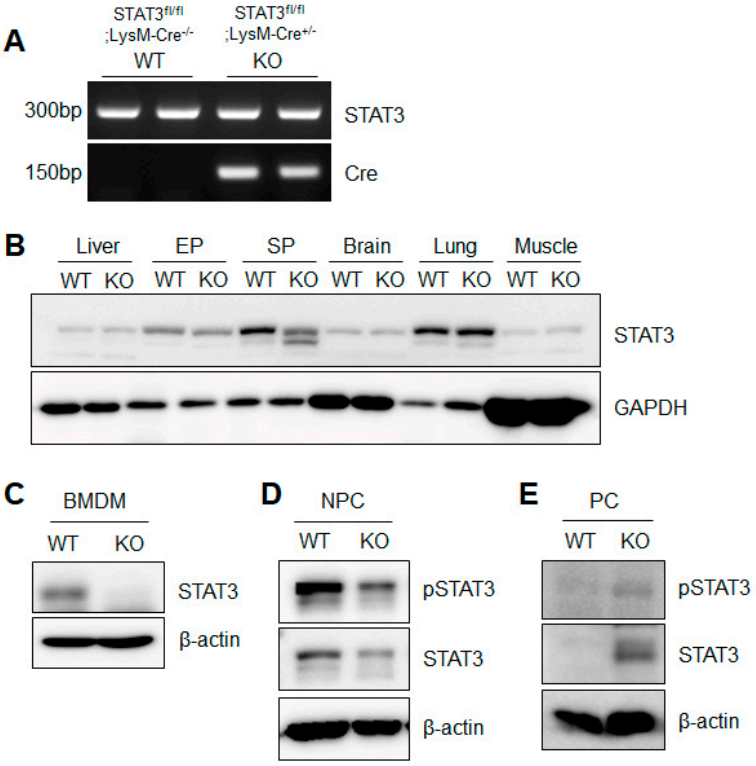

Figure S1. Identification of myeloid-specific STAT3-deficient mice. (A) PCR analyses for Cre and STAT3 alleles in genomic DNA from WT and mSTAT3KO mice. (B) STAT3 expression in the liver, epididymal fat pads (EP), spleen (SP), brain, lung, and skeletal muscle from WT and mSTAT3KO mice. (C) STAT3 expression in bone marrow-derived macrophages (BMDMs) from WT and mSTAT3KO mice. (D-E) Expression of pSTAT3 and STAT3 of isolated non-parenchymal cells (NPCs) (D) and parenchymal cells (PCs) (E) from WT and mSTAT3KO mice.

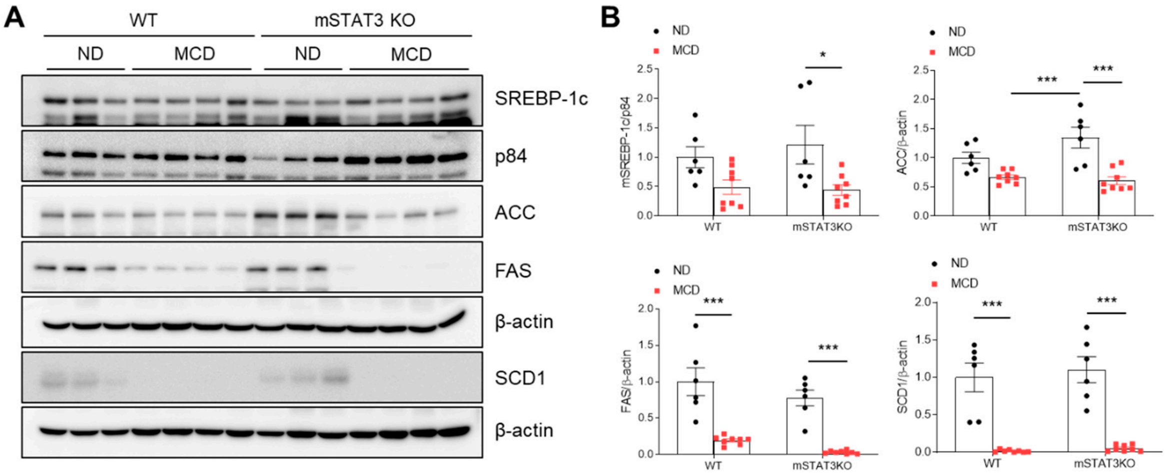

Figure S2. Effect of myeloid-specific STAT3 deletion on *de novo* lipogenesis in the livers of MCD diet-fed mice. **(**A-B) Western blot analysis and quantification of mature SREBP-1c (mSREBP-1c), ACC, FAS and SCD1 proteins in liver lysates (n = 6-8). Nuclear p84 and total β-actin were used as a loading control. Statistical significance was determined by two-way ANOVA. *$p < 0.05$, **$p < 0.01$, ***$p < 0.001$.
